# Comparative efficacy of statins, metformin, spironolactone and combined oral contraceptives in reducing testosterone levels in women with polycystic ovary syndrome: a network meta-analysis of randomized clinical trials

**DOI:** 10.1186/s12905-020-00919-5

**Published:** 2020-04-05

**Authors:** Hussain H. Almalki, Turki M. Alshibani, Abdullah A. Alhifany, Omar A. Almohammed

**Affiliations:** 1 Department of Clinical Pharmacy, College of Pharmacy, Umm Al-Qura University, 13578 Taif Rd, Makkah, 21955 Saudi Arabia; 2 Department of Clinical Pharmacy, College of Pharmacy, King Saud University, P.O. Box 2457, Riyadh, 11451 Saudi Arabia

**Keywords:** Statins, Metformin, Spironolactone, Contraceptives, PCOS, Testosterone

## Abstract

**Background:**

Polycystic ovary syndrome (PCOS) is an endocrine disorder affecting about 10% of women in reproductive age and associated with a variety of hormonal abnormalities, including hyperandrogenemia and infertility, all of which could lead to PCOS. Statins were previously introduced as a therapeutic option for reducing testosterone levels in women with PCOS, either alone or in combination. The aim of this study is to evaluate the effectiveness of different statins alone or in combination with metformin in reducing testosterone levels in women with PCOS.

**Methods:**

Medline, Embase, and clinicaltrials.gov were searched for studies that investigated the efficacy of statins, metformin, spironolactone, or combined oral contraceptives (COCs), individually or in combination, in reducing the testosterone level in patients with PCOS. The search was limited to randomized clinical trials and conducted according to the preferred reporting items for systematic reviews and meta-analyses - extension statement for network meta-analyses (PRISMA-NMA). The quality of included studies was assessed using the Cochrane Collaboration risk of bias (RoB) assessment tool. A frequentist network meta-analysis using random-effects models was used to assess the efficacy in reducing testosterone level and were expressed as odds ratios (OR) and 95% credible interval (95%Crl). All statistical analyses were performed using netmeta Version 1.0 on R statistical package.

**Result:**

Nine RCTs involving 613 patients were included. Atorvastatin showed greater reduction in testosterone level compared to COC (MD −2.78, 95%CrI −3.60, −1.97), spironolactone plus metformin (MD −2.83, 95%CrI −3.80, −1.87), simvastatin (MD −2.88, 95%CrI −3.85, −1.92), spironolactone (MD −2.90, 95%CI −3.77, −2.02), simvastatin plus metformin (MD −2.93, 95%CrI −3.79, −2.06), metformin (MD −2.97, 95%CrI −3.69, −2.25), lifestyle modification (MD −3.02, 95%CrI −3.87, −2.18), and placebo (MD −3.04, 95%CrI −3.56, −2.53).

**Conclusion:**

Atorvastatin was found to be more effective than the other management strategies in reducing the total testosterone level for patients with PCOS. Future studies should focus on the optimal dose.

## Introduction

Polycystic ovary syndrome (PCOS) is an endocrine disorder affecting about 10% of women in reproductive age and associated with a variety of hormonal abnormalities such as menstrual irregularity, insulin resistance, clinical hyperandrogenism or hyperandrogenemia, and infertility [1], all of which could lead to PCOS, despite their different features [2], The reduction in testosterone level is one of the most common outcomes reported in clinical trials [1–9], and it is frequently used by clinicians to assess the progress of patient’s condition.

The use of statins has been recently introduced as a therapeutic option for PCOS, either alone or in combination with metformin or combined oral contraceptives (COCs). To date, the guidelines recommend lifestyle modifications and weight reduction for all patients with PCOS along with COCs, metformin, or spironolactone to be individualized based on the patients’ presentation. However, the role of statins and its importance for patients with PCOS remains controversial [10].

COCs are the first-line therapies for managing menstrual irregularities in women with PCOS [4]. COCs exert their effect via reducing the level of androgen, which consequently lead to regulating menses [4]. For those intolerant to COCs, several trials confirmed the efficacy of metformin in reducing the androgen levels [3, 4, 6, 11]. Spironolactone inhibits the synthesis of steroids; therefore, it has been used in the treatment of PCOS to reduce high androgen-induced features, such as hirsutism and menstrual irregularity [9]. Simvastatin and atorvastatin are indicated for hypercholesterolemia [12] and have shown comparable efficacy to other antiandrogen agents in decreasing the level of androgen in women with PCOS [13].

In the absence of studies demonstrating the preferred therapy for patients with PCOS who present with high androgen-induced features, the current study was conducted to evaluate the effectiveness of different statins alone or in combination with metformin in treating patients with PCOS.

## Methods

A systematic review was conducted using Medline, Embase, and clinicaltrials.gov for studies using COCs, statins, spironolactone, and metformin for treatment of patients with PCOS. The patients, intervention, comparator, outcome, and study design (PICOS) strategy was used to identify relevant terms (Table 1), and search terms included polycystic ovary syndrome and antiandrogen. The search was limited to peer-reviewed randomized clinical trials (RCTs) that were conducted in humans and published in English. Studies were included if they evaluated the effect of medications on the change in total testosterone level as their efficacy outcome.

**Table 1:** PICOS framework

Population	Women with PCOS
Intervention	Statins
Comparator	Combined oral contraceptives (COC), metformin, placebo, lifestyle modification, or spironolactone
Outcome	Reduction in the blood level of testosterone
Study design	Published or unpublished randomized controlled trials

Data were extracted from the included studies by two independent investigators (HHA and TMA), and verified by a third investigator (AAA). For each study, the reduction in total testosterone reported as mean difference (MD) and standard deviation (SD) were extracted from studies as our primary outcome. If MD and SD were not provided in the studies, they were calculated using the equations in Table 2 [14]. A frequentist network meta-analysis using random-effects models was used to assess the efficacy in reducing testosterone levels and were expressed as odds ratio (OR) and 95% credible interval (95%Crl). All statistical analyses and inconsistency tests were performed using netmeta Version 1.0 on R statistical package [15]. The study was conducted according to the preferred reporting items for systematic reviews and meta-analyses for network meta-analyses (PRISMA-NMA) [16]. The risk of bias assessment was conducted for included studies using the Cochrane Collaboration risk of bias (RoB) assessment tool, and the Review Manager version 5.3 (Rev-man, the Nordic Cochrane Centre, Copenhagen 2014) were used to generate the RoB tables.

**Table 2:** Equations to calculate MD, SD, and correlation coefficient

Equation 1:	*Mean Change* (*MD*) = *M*2 − *M*1
Equation 2:	$SD\ Change=\sqrt{SD{1}^2+ SD{2}^2-\left(2\times Corr\times SD1\times SD2\right)}$
Equation 3:	$Corr=\frac{\left( SD{1}^2+ SD{2}^2- SD\ {change}^2\right)}{\left(2\times SD1\times SD2\right)}$

*M1*: Mean level of testosterone at baseline; *M2*: Mean level of testosterone after treatment, *SD1*: Standard deviation of testosterone level at baseline; *SD2*: standard deviation of testosterone level after treatment; *Corr*: correlation coefficient, which is the value that describes how similar the baseline and final measurements were across participant [14]

Equation 1 was used to calculate the mean difference of testosterone between baseline and after treatment, Equation 2 was used to impute a standard deviation of the change from baseline, and Equation 3: was used to calculate the correlation coefficient [14]. *Corr* was calculated using the *SD1*, *SD2*, and *SD Change*; since the SD change was not reported by some of the included studies, it was extrapolated from other similar studies [5, 8]

## Results

**Fig. 1:**
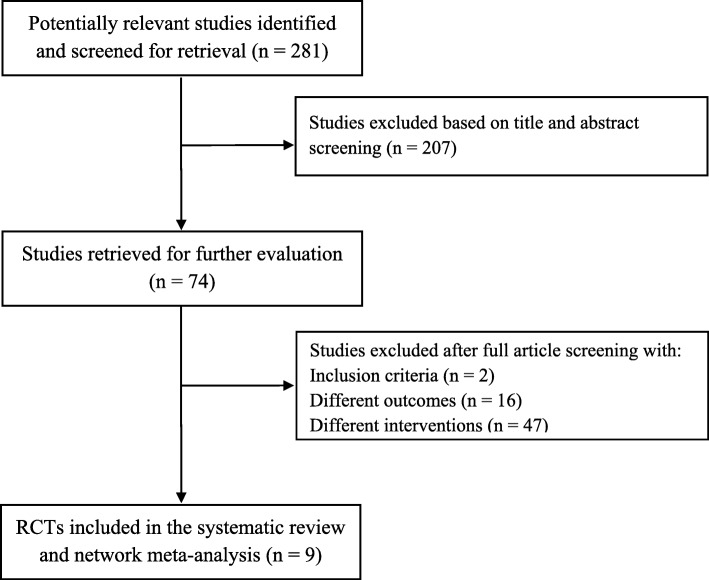
Flow diagram for study selection

A total of 281 articles were identified in the systematic search, and among these 9 articles were included in the network meta-analysis. The flowchart in Fig. 1 illustrates the process of including and excluding articles for this systematic review and network meta-analysis. The included studies were described in Table 3 [1–9].

**Table 3:** Studies that were included in the network meta-analysis

Author	Year	Country	Follow-up period	Design for the study	n	Age (yr.)	BMI (Kg/m^**2**^)	Intervention	Control
Ganie et al. [3]	2004	India	6 months	Randomized open-labelled clinical trial	69	17.6–28.5	20.9–32.1	Spironolactone (*n* = 34)	Metformin(*n* = 35)
Hoeger et al. [4]	2008	USA	24 weeks	Randomized clinical trial	43	13.7–17.7	27.8–46.0	Metformin (*n* = 10), COC (*n* = 11), or lifestyle modification (*n* = 11)	Placebo (*n* = 11)
Sathyapalan et al. [5]	2009	UK	12 weeks	Randomized double-blind clinical trial	37	26.3–29.1	31.8–35.4	Atorvastatin (*n* = 19)	Placebo (*n* = 18)
Kazerooni et al. [1]	2010	Iran	12 weeks	Randomized double-blind clinical trial	84	19.1–30.7	27.3–30.3	Metformin + Simvastatin (*n* = 42)	Metformin + Placebo (*n* = 42)
Romualdi et al. [6].	2010	Italy	6 months	Randomized double-blind clinical trial	28	20.3–29.8	18.4–26.2	Metformin (*n* = 15)	Placebo (*n* = 13)
Teede et al. [7]	2010	Australia	6 months	Randomized clinical trial	66	26.8–40.2	34.3–37.8	Metformin (*n* = 36)	COCs (*n* = 30)
Raja-Khan et al. [8]	2011	USA	6 weeks	Randomized double-blind clinical trial	20	23.6–38.1	25.6–51.9	Atorvastatin (*n* = 9)	Placebo (*n* = 11)
Banaszewska et al. [2]	2011	Poland	6 months	Randomized open-labelled clinical trial	97	24.7–26.9	22.9–25.6	Simvastatin + Metformin (*n* = 36)	Simvastatin (*n* = 28) or Metformin (*n* = 33)
Ganie et al. [9]	2013	India	6 months	Randomized open-labelled clinical trial	169	17.2–28.8	20.0–30.1	Spironolactone + Metformin (*n* = 62)	Spironolactone (*n* = 51) or Metformin (*n* = 56)

**Fig. 2:**
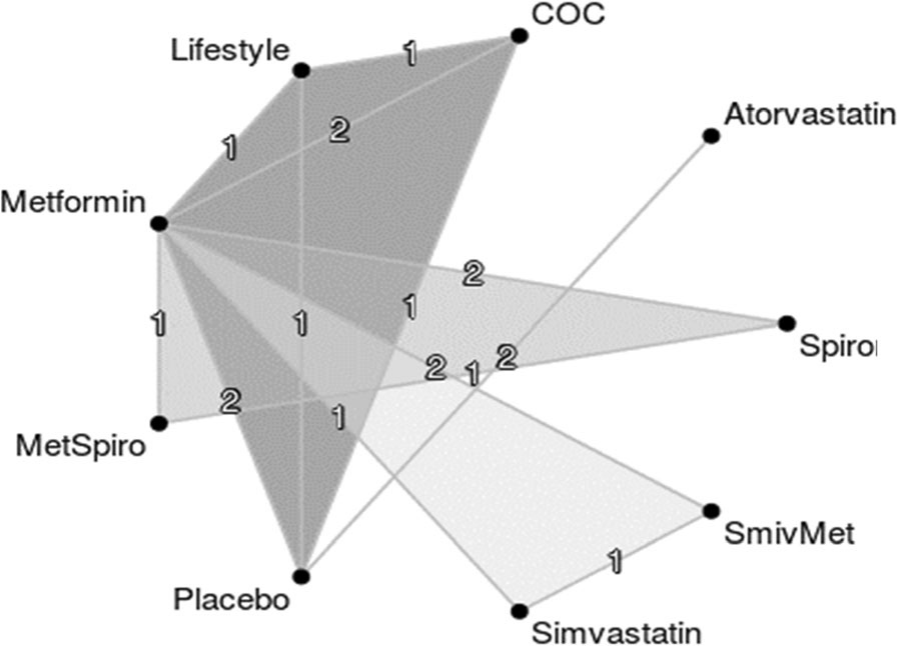
Network plot of all interventions in the analysis

The interventions in the included studies were COCs, atorvastatin, simvastatin, spironolactone, simvastatin plus metformin (SmivMet), metformin plus spironolactone (MetSpiro), metformin alone, and placebo. There was a direct comparison between atorvastatin and placebo and between simvastatin and metformin; however, no trials made a direct comparison between statins and other therapies. Therefore, a network meta-analysis deemed necessary to provide an indirect comparison between the interventions (Fig. 2). The summary of the results from the inconsistency test and the quality assessment of the included studies and risk of bias table were provided respectively in Table S1 and Figures S1 and S2 of the supplementary material.

### Comparative efficacy of interventions in PCOS

Metformin was the only management strategy that was evaluated directly to most of the other strategies. When compared to metformin, the pairwise comparison showed no significant difference in reducing the testosterone level for all management strategies. The results from the pairwise meta-analysis are presented in Table 4 above the leading diagonal.

**Table 4:** Network meta-analysis and pairwise meta-analysis of all interventions (Random Effect)

**Atorvastatin**								**−3.04 [− 3.56; − 2.53]**
**−2.78 [− 3.60; − 1.97]**	**COC**					− 0.18 [− 0.70; 0.34]	− 0.31 [− 1.08; 0.46]	− 0.35 [− 1.16; 0.46]
**−2.83 [− 3.80; − 1.87]**	− 0.05 [− 0.87; 0.78]	**MetSpiro**		− 0.09 [− 0.80; 0.62]	.	− 0.11 [− 0.80; 0.58]	.	.
**−2.88 [− 3.85; − 1.92]**	− 0.10 [− 0.92; 0.72]	−0.05 [− 0.96; 0.86]	**Simvastatin**	.	− 0.06 [− 0.74; 0.62]	−0.07 [− 0.75; 0.61]	.	.
**−2.90 [−3.77; − 2.02]**	−0.11 [− 0.83; 0.61]	−0.06 [− 0.72; 0.59]	−0.01 [− 0.83; 0.80]	**Spironolactone**	.	− 0.07 [− 0.58; 0.43]	.	.
**−2.93 [− 3.79; − 2.06]**	−0.14 [− 0.85; 0.56]	−0.09 [− 0.90; 0.72]	−0.04 [− 0.68; 0.60]	−0.03 [− 0.73; 0.67]	**SimvMet**	−0.04 [− 0.53; 0.44]	.	.
**−2.97 [−3.69; − 2.25]**	−0.19 [− 0.70; 0.33]	−0.14 [− 0.78; 0.51]	−0.09 [− 0.73; 0.55]	−0.07 [− 0.58; 0.43]	−0.04 [− 0.53; 0.44]	**Metformin**	−0.02 [− 0.70; 0.67]	−0.05 [− 0.56; 0.47]
**−3.02 [− 3.87; − 2.18]**	−0.24 [− 0.93; 0.45]	−0.19 [− 1.09; 0.71]	−0.14 [− 1.03; 0.75]	−0.13 [− 0.93; 0.67]	−0.10 [− 0.89; 0.69]	−0.05 [− 0.68; 0.57]	**Lifestyle**	−0.03 [− 0.76; 0.70]
**− 3.04 [− 3.56; − 2.53]**	−0.26 [− 0.89; 0.38]	−0.21 [− 1.03; 0.61]	−0.16 [− 0.97; 0.65]	−0.15 [− 0.86; 0.56]	−0.12 [− 0.81; 0.58]	−0.07 [− 0.57; 0.43]	−0.02 [− 0.68; 0.65]	**Placebo**

Treatments were ranked from best to least along the leading diagonal from the random-effect analysis. Above the leading diagonal are estimates from pairwise meta-analyses and below are estimates from the network meta-analyses. Data presented are mean difference (MD) with 95% confidence interval (95%CI) or 95% credible interval (95%CrI) for the pairwise meta-analysis or network meta-analysis, respectively. MD below 0 indicate higher efficacy in reducing testosterone level. Bold values indicate comparisons that are statistically significant

*Abbreviations*: *COC* combined oral contraceptive, *MetSpiro* Spironolactone plus Metformin, *SimvMet* Metformin plus Simvastatin

In the network meta-analysis, atorvastatin showed greater reduction in testosterone level compared to COC (MD −2.78, 95%CrI −3.60, − 1.97), spironolactone plus metformin (MD −2.83, 95%CrI −3.80, −1.87), simvastatin (MD −2.88, 95%CrI −3.85, −1.92), spironolactone (MD −2.90, 95%CI −3.77, −2.02), simvastatin plus metformin (MD −2.93, 95%CrI −3.79, −2.06), metformin (MD −2.97, 95%CrI −3.69, −2.25), lifestyle modification (MD −3.02, 95%CrI −3.87, −2.18), and placebo (MD −3.04, 95%CrI −3.56, −2.53). The results from network meta-analysis is presented in Table 4 below the leading diagonal.

## Discussion

The study evaluated the effect of atorvastatin in reducing testosterone levels in women with PCOS utilizing the network-meta analysis technique to provide a direct and indirect comparison of all interventions used to treat PCOS, with the goal of providing a comprehensive picture of statins alone or as add-on therapy with metformin or COC for clinicians and patients. The study found that atorvastatin provided a greater reduction in testosterone levels in patients with PCOS when compared to COC, spironolactone plus metformin, simvastatin, spironolactone, simvastatin plus metformin, metformin, lifestyle modification, and placebo, respectively.

The pros and cons of atorvastatin use in improving PCOS symptoms have been addressed in the literature [17–19]. It has been shown that using atorvastatin for a duration of more than 12 weeks had substantially improved PCOS symptoms [17], yet, it impairs insulin sensitivity [18]. However, to our knowledge, this is the first study to compare between atorvastatin and all interventions in terms of reducing the testosterone level in patients with PCOS. Initially, we have combined atorvastatin and simvastatin as one group (statins); however, this led to a significant inconsistency (*p*-value < 0.05). Hence, we have separated the atorvastatin and the simvastatin into two groups.

COC is the first line of treatment in patients with PCOS as it provides a great remission for PCOS symptoms [10]; however, it prevents patients from conceiving, if they want to be. Metformin alone, as the first line of treatment, allows patients to conceive, but it is inferior in terms of resolving PCOS symptoms [10]. From the current study, atorvastatin was found to be better in reducing testosterone levels, which would resolve PCOS symptoms without interfering with the ability to conceive.

The main limitations of our network meta-analysis are the significant heterogeneity among the included studies. The nine studies that were included in the NMA used different diagnostic criteria for PCOS, leading to different types of participants were included. Also, different types and doses of statins were used, which could have affected the outcome of the study. The baseline characteristics for the participants in the trials differed in terms of age, BMI, and ethnicity, which may have affected the results. Moreover, the design for the studies, drug dosage, and follow-up duration, thus it may have affected the results. Finally, in the current study, we only evaluated the interventions based on the change in the testosterone level, which might limit the utilization of the results to patients with a high level of testosterone only.

## Conclusion

The findings from the current study supports the use of atorvastatin over COC, spironolactone plus metformin, simvastatin, spironolactone, simvastatin plus metformin, metformin, lifestyle modification, and placebo, as it was associated with the greatest reduction in testosterone levels; knowing that the anaylyses were conducted including the beset available evidence at the time of the study. Therefore, atorvastatin should be recommended, with caution, in PCOS patients who present with a high level of testosterone. Larger randomized clinical trials are needed to identify atorvastatin dose with the best effect in patients with PCOS.

## Supplementary information

**Additional file 1 Table S1.** Direct and indirect comparison from the network meta-analysis and the inconsistency test results

**Additional file 2 Figure S1**. Summary for the risk of bias assessment

**Additional file 3 Figure S2**. Risk of bias table of all included studies.

## Data Availability

All data generated or analyzed during this study are included in this published article [and its supplementary information files].

## Abbreviations

PCOS: Polycystic ovary syndrome
COCs: Combined oral contraceptives
RoB: Risk of bias
OR: Odds ratios
95%Crl: 95% Credible interval
MD: Mean difference
SD: Standard deviation
Corr: Correlation coefficient
SmivMet: Simvastatin plus metformin
MetSpiro: Metformin plus spironolactone
