# Supplementary material for: Comparative efficacy of statins, metformin, spironolactone and combined oral contraceptives in reducing testosterone levels in women with polycystic ovary syndrome: a network meta-analysis of randomized clinical trials
Source: BMC Womens Health. 2020 Apr 5;20:68. doi: 10.1186/s12905-020-00919-5 (PMC7132972; doi:10.1186/s12905-020-00919-5)
Supplement: Supplementary file 1 — Additional file 1 Table S1. Direct and indirect comparison from the network meta-analysis and the inconsistency test results [file 12905_2020_919_MOESM1_ESM.docx]

| Table S1: Direct and indirect comparison from the network meta-analysis and the inconsistency test results | | | | | | | | |
| --- | --- | --- | --- | --- | --- | --- | --- | --- |
| **Comparison** | **No. of Studies** | **NMA** | **Direct** | **Indirect** | **Difference** | **Diff. lower 95%CI** | **Diff. upper 95%CI** | ***p*-value** |
| Atorvastatin:COC | 0 | -2.78 | NA | -2.78 | NA | NA | NA | NA |
| Atorvastatin:Lifestyle | 0 | -3.02 | NA | -3.02 | NA | NA | NA | NA |
| Atorvastatin:Metformin | 0 | -2.97 | NA | -2.97 | NA | NA | NA | NA |
| Atorvastatin:MetSpiro | 0 | -2.83 | NA | -2.83 | NA | NA | NA | NA |
| Atorvastatin:Placebo | 2 | -3.04 | -3.04 | NA | NA | NA | NA | NA |
| Atorvastatin:Simvastatin | 0 | -2.88 | NA | -2.88 | NA | NA | NA | NA |
| Atorvastatin:SmivMet | 0 | -2.93 | NA | -2.93 | NA | NA | NA | NA |
| Atorvastatin:Spironolactone | 0 | -2.90 | NA | -2.90 | NA | NA | NA | NA |
| COC:Lifestyle | 1 | -0.24 | -0.31 | 0.05 | -0.36 | -2.06 | 1.34 | 0.68 |
| COC:Metformin | 2 | -0.19 | -0.18 | -0.32 | 0.14 | -2.38 | 2.66 | 0.91 |
| COC:MetSpiro | 0 | -0.05 | NA | -0.05 | NA | NA | NA | NA |
| COC:Placebo | 1 | -0.26 | -0.35 | -0.12 | -0.23 | -1.53 | 1.07 | 0.73 |
| COC:Simvastatin | 0 | -0.10 | NA | -0.10 | NA | NA | NA | NA |
| COC:SmivMet | 0 | -0.14 | NA | -0.14 | NA | NA | NA | NA |
| COC:Spironolactone | 0 | -0.11 | NA | -0.11 | NA | NA | NA | NA |
| Lifestyle:Metformin | 1 | 0.05 | 0.02 | 0.22 | -0.20 | -1.82 | 1.43 | 0.81 |
| Lifestyle:MetSpiro | 0 | 0.19 | NA | 0.19 | NA | NA | NA | NA |
| Lifestyle:Placebo | 1 | -0.02 | -0.03 | 0.07 | -0.10 | -1.89 | 1.70 | 0.91 |
| Lifestyle:Simvastatin | 0 | 0.14 | NA | 0.14 | NA | NA | NA | NA |
| Lifestyle:SmivMet | 0 | 0.10 | NA | 0.10 | NA | NA | NA | NA |
| Lifestyle:Spironolactone | 0 | 0.13 | NA | 0.13 | NA | NA | NA | NA |
| MetSpiro:Metformin | 1 | -0.14 | -0.11 | -0.33 | 0.22 | -1.79 | 2.23 | 0.83 |
| Placebo:Metformin | 2 | 0.07 | 0.05 | 0.54 | -0.49 | -2.82 | 1.84 | 0.68 |
| Simvastatin:Metformin | 1 | -0.09 | -0.07 | -0.21 | 0.14 | -1.80 | 2.08 | 0.89 |
| SmivMet:Metformin | 2 | -0.04 | -0.04 | NA | NA | NA | NA | NA |
| Spironolactone:Metformin | 2 | -0.07 | -0.07 | NA | NA | NA | NA | NA |
| MetSpiro:Placebo | 0 | -0.21 | NA | -0.21 | NA | NA | NA | NA |
| MetSpiro:Simvastatin | 0 | -0.05 | NA | -0.05 | NA | NA | NA | NA |
| MetSpiro:SmivMet | 0 | -0.09 | NA | -0.09 | NA | NA | NA | NA |
| MetSpiro:Spironolactone | 1 | -0.06 | -0.09 | 0.12 | -0.21 | -2.13 | 1.71 | 0.83 |
| Placebo:Simvastatin | 0 | 0.16 | NA | 0.16 | NA | NA | NA | NA |
| Placebo:SmivMet | 0 | 0.12 | NA | 0.12 | NA | NA | NA | NA |
| Placebo:Spironolactone | 0 | 0.15 | NA | 0.15 | NA | NA | NA | NA |
| Simvastatin:SmivMet | 1 | -0.04 | -0.06 | 0.08 | -0.14 | -2.08 | 1.80 | 0.89 |
| Simvastatin:Spironolactone | 0 | -0.01 | NA | -0.01 | NA | NA | NA | NA |
| SmivMet:Spironolactone | 0 | 0.03 | NA | 0.03 | NA | NA | NA | NA |
