# Supplementary material for: Comparative efficacy of statins, metformin, spironolactone and combined oral contraceptives in reducing testosterone levels in women with polycystic ovary syndrome: a network meta-analysis of randomized clinical trials
Source: BMC Womens Health. 2020 Apr 5;20:68. doi: 10.1186/s12905-020-00919-5 (PMC7132972; doi:10.1186/s12905-020-00919-5)
Supplement: Supplementary file 2 — Additional file 2 Figure S1. Summary for the risk of bias assessment [file 12905_2020_919_MOESM2_ESM.docx]

Figure S1. Summary for the risk of bias assessment

This figure summarizes the risk of bias for each study as a risk of bias summary of the overall meta-analysis. Green represents low risk of bias and red represents high risk of bias.
