# Supplementary material for: Comparative efficacy of statins, metformin, spironolactone and combined oral contraceptives in reducing testosterone levels in women with polycystic ovary syndrome: a network meta-analysis of randomized clinical trials
Source: BMC Womens Health. 2020 Apr 5;20:68. doi: 10.1186/s12905-020-00919-5 (PMC7132972; doi:10.1186/s12905-020-00919-5)
Supplement: Supplementary file 3 — Additional file 3 Figure S2. Risk of bias table of all included studies. [file 12905_2020_919_MOESM3_ESM.docx]

Figure S2. Risk of bias table of all included studies.

(+) denotes low risk of bias, blank denotes unclear risk of bias, and (−) denotes high risk of bias.
